# Biological Effects of Corticosteroids on Pneumococcal Pneumonia in Mice and Humans

**DOI:** 10.21203/rs.3.rs-3962861/v1

**Published:** 2024-02-21

**Authors:** Hiroki Taenaka, Katherine D. Wick, Aartik Sarma, Shotaro Matsumoto, Rajani Ghale, Xiaohui Fang, Mazharul Maishan, Jeffrey E. Gotts, Charles R. Langelier, Carolyn S. Calfee, Michael A. Matthay

**Affiliations:** University of California, San Francisco; Mayo Clinic; University of California, San Francisco; Tokyo Medical and Dental University; University of California, San Francisco; University of California, San Francisco; University of California, San Francisco; University of California, San Francisco; University of California, San Francisco; University of California, San Francisco; University of California, San Francisco

**Keywords:** Pneumonia, Acute respiratory distress syndrome, Glucocorticoids, Streptococcal infections

## Abstract

**Background:**

*Streptococcus pneumoniae* is the most common bacterial cause of community acquired pneumonia and the acute respiratory distress syndrome (ARDS). Some clinical trials have demonstrated a beneficial effect of corticosteroid therapy in community acquired pneumonia, COVID-19, and ARDS, but the mechanisms of this benefit remain unclear. The objective of this study was to investigate the effects of corticosteroids on the pulmonary biology of pneumococcal pneumonia in an observational cohort of mechanically ventilated patients and in a mouse model of bacterial pneumonia with *Streptococcus pneumoniae*.

**Methods:**

We studied gene expression with lower respiratory tract transcriptomes from a cohort of mechanically ventilated patients and in mice. We also carried out comprehensive physiologic, biochemical, and histological analyses in mice to identify the mechanisms of lung injury in *Streptococcus pneumoniae* with and without adjunctive steroid therapy.

**Results:**

Transcriptomic analysis identified pleiotropic effects of steroid therapy on the lower respiratory tract in critically ill patients with pneumococcal pneumonia, findings that were reproducible in mice. In mice with pneumonia, dexamethasone in combination with ceftriaxone reduced (1) pulmonary edema formation, (2) alveolar protein permeability, (3) proinflammatory cytokine release, (4) histopathologic lung injury score, and (5) hypoxemia but did not increase bacterial burden.

**Conclusions:**

The gene expression studies in patients and in the mice support the clinical relevance of the mouse studies, which replicate several features of pneumococcal pneumonia and steroid therapy in humans. In combination with appropriate antibiotic therapy in mice, treatment of pneumococcal pneumonia with steroid therapy reduced hypoxemia, pulmonary edema, lung permeability, and histologic criteria of lung injury, and also altered inflammatory responses at the protein and gene expression level. The results from these studies provide evidence for the mechanisms that may explain the beneficial effects of glucocorticoid therapy in patients with community acquired pneumonia from *Streptococcus Pneumoniae*.

## INTRODUCTION

Bacterial pneumonia is a frequent cause of severe respiratory failure and acute respiratory distress syndrome (ARDS) [1]. The most common cause of bacterial community acquired pneumonia (CAP) is *Streptococcus pneumoniae* [2, 3], which directly damages the lung epithelium and induces the release of a substantial number of cytokines and chemokines from epithelial cells and macrophages, resulting in protein exudation and edema formation in the lung [1, 4]. Despite effective antibiotic therapy, the dysregulated pathogen-host interaction may still cause life-threatening acute respiratory failure [5].

Corticosteroids are sometimes prescribed as an adjunctive therapy for severe pulmonary infections and ARDS. The clinical benefit of adjunctive steroid therapy for pneumococcal infections has been previously demonstrated in meningitis [6]. Recent clinical trials have demonstrated therapeutic benefits of dexamethasone, a long-acting glucocorticoid, on mortality in severe COVID-19 infection [7], non-COVID ARDS [8], and in severe CAP [9–11]. However, the mechanisms by which steroids improve outcomes in patients with severe pneumonia or ARDS are not well established, and few studies have comprehensively characterized the effects of steroids in the respiratory tract of critically ill patients [12, 13] or clinically relevant animal models using bacterial pathogens treated with antibiotic therapy [14, 15].

Corticosteroids are typically used to potently suppress systemic immune responses, but they have additional effects that may be relevant to lung injury, including effects on wound healing, modifying lung fluid balance and the extent of pulmonary edema, and metabolism [16, 17]. There is emerging evidence that corticosteroids have distinct effects on systemic *vs*. pulmonary inflammation [18, 19]. Given that corticosteroids have pleiotropic and lung-specific effects, elucidating the effects of corticosteroids on the injured lung in bacterial pneumonia is important for establishing a biologic rationale for further clinical application of steroids in severe lung infections. Understanding the specific mechanisms of corticosteroid benefit should help identify the patients with pneumonia or ARDS most likely to benefit from steroids.

To investigate the effects of corticosteroids in bacterial pneumonia, we studied lower respiratory tract gene expression in an observational cohort of mechanically ventilated patients with and without *S. pneumoniae* pneumonia and in mice with an established mouse model of pneumococcal pneumonia. We also carried out comprehensive biochemical, physiological, and histologic studies in the mice to understand the effects of steroids in pneumococcal pneumonia.

## METHODS

*See* the online supplement for more detailed methods.

### Observational cohort

**Figure 1:**
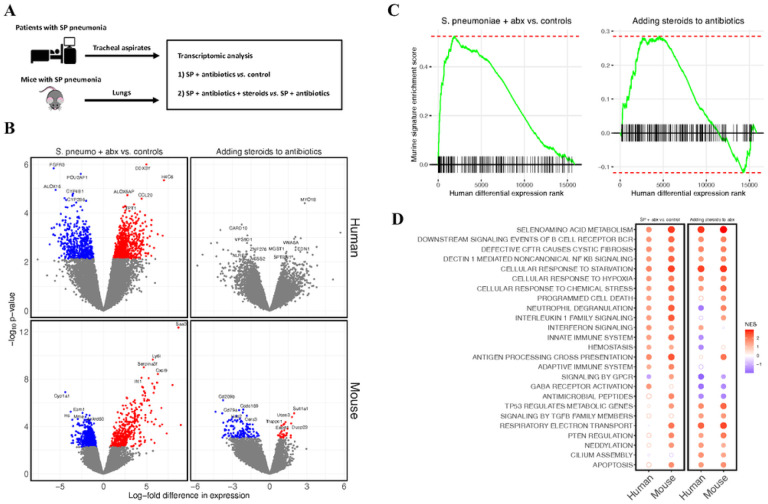
RNA sequencing identifies reproducible effects of pneumococcal pneumonia and steroid treatment in humans and mice. (*A*) Study design. Tracheal aspirate samples were collected from an observational cohort of mechanically ventilated adults within 72 hours of admission to the ICU. Patients who had *Streptococcus pneumoniae* (SP) in a respiratory tract culture or met criteria for SP infection using an established metagenomic sequencing classifier model. Lungs were collected from mouse model of SP pneumonia. (*B*) Volcano plots of differential gene expression. Each point represents an individual gene. The log_2_-fold difference in gene expression estimated by limma for each comparison is on the *x*-axis, and the p-value for each comparison is on the y axis. A positive difference in gene expression indicates the gene is more highly expressed in humans or mice with *S. pneumoniae* treated with antibiotics in the first column, and more highly expressed in humans or mice who received steroids in the second column. Grey points were not statistically significant (FDR < 0.1) after adjusting for multiple hypothesis testing. (*C*) Enrichment plots for mouse gene signatures in human differential expression. Genes were ranked along the x-axis by the estimated log-fold difference in gene expression between groups in human tracheal aspirate transcriptomes. A vertical line along the *x*-axis identifies the genes that are present in the mouse gene signature. The green line represents the running GSEA enrichment score at that position on the ranked gene list. (*D*) Dot plot of GSEA net enrichment scores (NES) for the differential expression data in panel B. Red indicates genes in selected Reactome pathways that are relatively more highly expressed in humans or mice with *S. pneumoniae* treated with antibiotics in first and second columns, and more highly expressed in humans or mice who received steroids in the third and fourth columns. A solid circle indicates GSEA FDR <0.1. ***Abbreviations*:** SP *streptococcus pneumoniae*; NES net enrichment score.

We studied tracheal aspirate gene expression from samples collected from an observational cohort of mechanically ventilated adults within 72 hours of admission to the ICU at UCSF Medical Center. The study was approved by the UCSF Institutional Review Board (17–24056). We included all subjects who had *S. pneumoniae* in a respiratory tract culture or met criteria for *S. pneumoniae* infection using a metagenomic sequencing-based model as previously described [20]. We also included as controls subjects who were mechanically ventilated for neurologic injury, were not immunocompromised, received no immunosuppressive medications, and had no evidence of respiratory disease on chest radiograph (Fig. 1A).

#### Experimental Model in Mice, Bacterial Infection, and Treatment:

**Figure 2:**
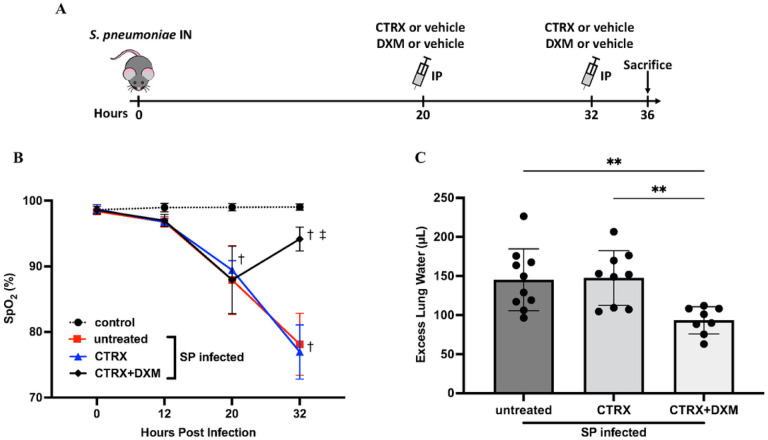
Dexamethasone prevents the progression of hypoxemia and pulmonary edema in mice with pneumococcal pneumonia. (*A*) Mice were inoculated intranasally (IN) with 10^8^ colony-forming units of Streptococcus pneumoniae (SP) and received 10mg/kg of dexamethasone or vehicle control in conjunction with 150 mg/kg of ceftriaxone or vehicle control intraperitoneally (IP) 20 and 32 hours after bacterial inoculation. Mice were sacrificed 36 hours after infection. (*B*) Arterial oxygen saturation (SpO_2_) was measured 12, 20, and 32 hours after bacterial inoculation. SpO_2_ in the infected mice declined 20 hours after infection. Dexamethasone-treated mice had higher SpO_2_ 32 hours after infection than mice without dexamethasone. n = 5/group. (*C*) Excess lung water, a measure of edema in the interstitial and alveolar spaces, were reduced with combination treatment of dexamethasone and ceftriaxone when compared to untreated mice and ceftriaxone alone. n = 7–10/group. All data are shown as means ± SD. Statistical differences between groups were calculated with (*B*) two-way repeated ANOVA followed by Tukey’s multiple comparison test, or (*C*) one-way ANOVA followed by Tukey’s multiple comparison test. ^†^P < 0.05 compared with control. ^‡^ P < 0.05 compared with groups without dexamethasone. **P < 0.01 ***Abbreviations*:** IN intranasal injection; IP intraperitoneal injection; CTRX ceftriaxone; DXM dexamethasone SpO_2_; arterial oxygen saturation; SP *streptococcus pneumoniae*.

The protocol was approved by the University of California, San Francisco Institutional Animal Care and Use Committee (No.AN189182). C57BL/6 mice were randomly divided into four groups: (1) Healthy control; (2) *S. pneumoniae*; (3) *S. pneumoniae* + ceftriaxone; (4) *S. pneumoniae* + ceftriaxone + dexamethasone. Mice in groups 2–4 were anesthetized and inoculated intranasally with 10^8^ CFU of live *S. pneumoniae* serotype 19F. Mice received 10 mg/kg of dexamethasone or vehicle control in combination with 150 mg/kg of ceftriaxone or vehicle control intraperitoneally 20 and 32 hours after inoculation. In some experiments, body temperature, body weight, and oxygen saturation by pulse oximetry (SpO_2_) were measured 12, 20, and 32 hours after inoculation. All mice were sacrificed 36 hours after infection or earlier if pre-specified criteria indicating unacceptably severe illness were met. Figure 2A depicts experimental procedures.

### Lung Injury Endpoints

Mice underwent overdose of ketamine and xylazine, bilateral thoracotomy, and exsanguination by right ventricular puncture. In some mice, lungs were removed and homogenized to measure excess extravascular lung water (ELW) [21, 22]. In other mice, the lungs were lavaged, and bronchoalveolar lavage (BAL) protein was measured with the BCA Protein Assay (Thermo Fisher). BAL cell count was measured with a Coulter counter. Postmortem bacterial titers of BAL were measured by serial dilution and plaque counting on sheep blood agar plates. For histology, the lungs were fixed by 4% paraformaldehyde and stained with hematoxylin-eosin. Lung injury was assessed by an observer (XF) who was blinded to the experimental groups, based on the scoring system partly modified from the report of the American Thoracic Society [23]. BAL inflammatory cytokines and chemokines were measured using a FLEXMAP 3D^™^ (Luminex). Receptor for advanced glycation end products (RAGE) in BAL was measured using the Quantikine ELISA kit (R&D Systems). Alveolar fluid clearance was measured according to previously published methods [24, 25].

### RNA Sequencing

In mice, the whole lung was placed in RNA Shield, frozen, homogenized, and then samples extracted using Quick-RNA mini prep plus kit (Zymo research). In humans, tracheal aspirate samples were collected on the day of enrollment and stored at −80C in RNAse-free conditions [26]. RNA was extracted using the Allprpep kit (Qiagen and then ribodepleted using FastSelect (Qiagen) before undergoing library preparation using the NEBNext Ultra II RNASeq Kit (New England Biolabs) and paired-end Illumina sequencing on an Novaseq 6000. Human and mouse samples were sequenced separately.

### Bioinformatics analysis

Transcriptomic analysis was performed using established bioinformatics pipelines. Briefly, FASTQ files were aligned using STAR and count matrices were generated using *tximport*. Differential expression between groups was determined using *limma-voom*, and fgsea was used to identify pathways enriched in each experimental condition. *CEMiTool* was used to perform an unsupervised network analysis. A Benjamini-Hochberg adjusted p-value < 0.1 was considered significant for all analyses. Full details are provided in online supplement.

### Statistical analysis

All data were tested for normality with Shapiro-Wilk tests. Comparisons of each group were made with one-way ANOVA followed by Tukey’s multiple comparisons tests for normally distributed parameters, or with Kruskal-Wallis test followed by Dunn’s multiple comparisons for skewed parameters. Two-way analysis of variance for repeated measures followed by Tukey’s multiple comparison test was used to evaluate the effect of time and group. All tests were two-tailed, and differences were considered to be statistically significant when p < 0.05.

## RESULTS

### Human subjects

Tracheal aspirate transcriptomes were available from 159 mechanically ventilated subjects, of whom 15 met inclusion criteria. Ten of the patients had clinically adjudicated *S. pneumoniae* pneumonia, in whom five the pathogen was detected by culture and five by metagenomic sequencing [20, 27] (**Table S1**). All patients were treated with antibiotics active against *S. pneumoniae* prior to sample collection, and five patients (three culture-positive) also received corticosteroids prior to sample collection. Samples were also available from five uninfected control subjects.

#### RNA sequencing identifies reproducible effects of both pneumococcal pneumonia and steroid treatment in human patients and mice

*S. pneumoniae* infection was associated with markedly altered gene expression in the lower respiratory tracts of both humans and mice (Fig. 1B). There were 1,184 genes differentially expressed between patients with *S. pneumoniae* who were not treated with steroids versus uninfected controls, and 951 genes differentially expressed between infected mice treated with ceftriaxone and uninfected controls. No individual gene was differentially expressed between pneumonia patients who received steroids versus those who did not, while 162 genes were differentially expressed in mice treated with dexamethasone and ceftriaxone compared to mice treated with ceftriaxone alone (Fig. 1B). Gene set enrichment analysis (GSEA) identified notable overlaps between gene expression from clinical samples and gene expression in the mouse model (Fig. 1C), suggesting that the experimental model replicated biological features of clinical disease and steroid treatment. The top 100 genes upregulated in patients with *S. pneumoniae* pneumonia compared to controls were significantly enriched in the *S. pneumoniae* mouse model compared to control mice (p = 1.2×10^− 5^). The top 100 genes upregulated in patients with pneumococcal pneumonia who received steroids compared to patients with pneumococcal pneumonia who did not receive steroids were significantly enriched in mice treated with dexamethasone and ceftriaxone compared to mice treated with ceftriaxone alone (p = 1.9×10^− 12^).

GSEA identified enrichment of several pathways shared between humans and mice (Fig. 1D). For example, in both mice and humans, *S. pneumoniae* infection, as expected, increased expression of cellular stress responses and upregulated adaptive and innate immune signaling pathways, including interferon and interleukin 1 family signaling, neutrophil degranulation, and B cell receptor signaling compared to uninfected controls. The addition of steroids to antibiotics increased the expression of pathways associated with injury resolution, including TGF-beta signaling, anti-inflammatory signaling by PTEN, and cilium assembly pathways in humans and in mice. Interestingly, several pathways that were more highly expressed in *S. pneumoniae* infection compared to controls, including stress and hypoxia responses, non-canonical NF-kB signaling, and B cell receptor signaling were increased further by the addition of steroids. Weighted gene co-expression network analysis (WGCNA) results are described in the **Supplementary Material**.

### Dexamethasone prevents the progression of hypoxemia and pulmonary edema in mice with pneumococcal pneumonia

#### Oxygen Saturation.

Mice inoculated intranasally with 10^8^ CFU of *S. pneumoniae* developed hypoxemia at 20 hours after infection. The average oxygen saturation (SpO_2_) declined to approximately 87–91% (Fig. 2B). At 20 hours, mice received either no treatment, ceftriaxone, or ceftriaxone plus dexamethasone. By 32 hours, dexamethasone-treated mice had significantly higher oxygen saturation than those who did not receive dexamethasone. Thus, dexamethasone treatment attenuated progression of arterial hypoxemia 12 hours after treatment, whereas ceftriaxone treatment did not prevent the progression of hypoxemia. Note that arterial hypoxemia in the mice treated with antibiotics alone reached a mean value of 76% at 32 hours whereas dexamethasone treatment increased oxygen saturation to a mean level above 90%.

#### Pulmonary Edema.

Mice inoculated with *S. pneumoniae* had approximately 150μL of excess lung water (ELW), demonstrating that 10^8^ of *S. pneumoniae* induced substantial pulmonary edema within 36 hours after infection. The results for hypoxemia and ELW by treatment group were similar, underscoring the relevance of ELW as a marker of physiologically important pulmonary edema (Fig. 2A, B). Treatment with ceftriaxone alone did not reduce ELW. However, the combination of ceftriaxone and dexamethasone significantly reduced ELW compared to the untreated mice and to ceftriaxone alone (Fig. 2B).

#### Temperature and Body Weight.

Infected mice became hypothermic (approximately 30°C) at 12 hours after infection and had lower body temperature than normal healthy control mice throughout the experiment. Mice in all infected groups had significant body weight loss compared with normal healthy controls. (**Figure S3**).

### Dexamethasone reduces both alveolar protein permeability and accumulation of leukocytes in the airspaces

**Figure 3:**
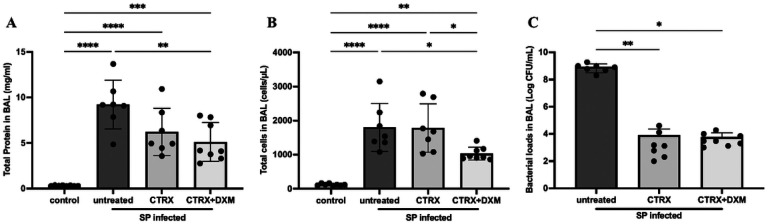
Dexamethasone reduces both alveolar protein permeability and cell infiltration. (*A*) Total protein concentration in bronchoalveolar lavage (BAL) fluid were elevated in the infected mice, indicating alveolar-capillary barrier disruption. The combination of dexamethasone and ceftriaxone reduced the BAL protein concentration, whereas ceftriaxone alone did not. n = 7–8/group. (*B*) Total cell number in BAL fluid was elevated in infected mice. The combination of dexamethasone and ceftriaxone reduced the total cell number compared with untreated and ceftriaxone alone. n = 7–8/group. (*C*) Ceftriaxone substantially reduced the bacterial loads 36 hours after infection. The addition of dexamethasone to appropriate antibiotic treatment with ceftriaxone did not increase bacterial loads. n = 7–8/group. All data are shown as means ± SD. Statistical differences between groups were calculated with one-way ANOVA followed by Tukey’s multiple comparison test or Kruskal-Wallis followed by Dunn’s multiple comparison. *P < 0.05, **P < 0.01, ***P < 0.001, ****P < 0.0001. ***Abbreviations*:** CFU colony forming unit; BAL bronchoalveolar lavage; SP *streptococcus pneumoniae*; CTRX ceftriaxone; DXM dexamethasone.

Mice with *S. pneumoniae* had higher protein concentrations in BAL compared with controls, indicating alveolar-capillary barrier disruption (Fig. 3A). The combination of ceftriaxone and dexamethasone significantly decreased BAL protein concentration by one way ANOVA; ceftriaxone alone also decreased BAL protein concentration, though not significantly compared to untreated mice. Total cell counts in BAL showed a substantial increase of white blood cells in the distal airpaces with *S. pneumoniae* (Fig. 3B). Similarly to the pulmonary edema results, treatment with ceftriaxone alone did not reduce total leukocyte accumulation. However, the combination of ceftriaxone and dexamethasone significantly reduced the accumulation of white blood cells compared to untreated mice and to ceftriaxone alone. Most of the leukocytes were neutrophils (Figure S4).

#### Bacterial load in the bronchoalveolar lavage (BAL).

Since dexamethasone may impair host defense, we measured the bacterial load in BAL (Fig. 3C). Ceftriaxone substantially reduced the bacterial loads 36 hours after inoculation, as expected. The addition of dexamethasone to ceftriaxone treatment did not increase the bacterial burden in the BAL.

### Dexamethasone improves lung histopathology in mice with pneumococcal pneumonia

**Figure 4:**
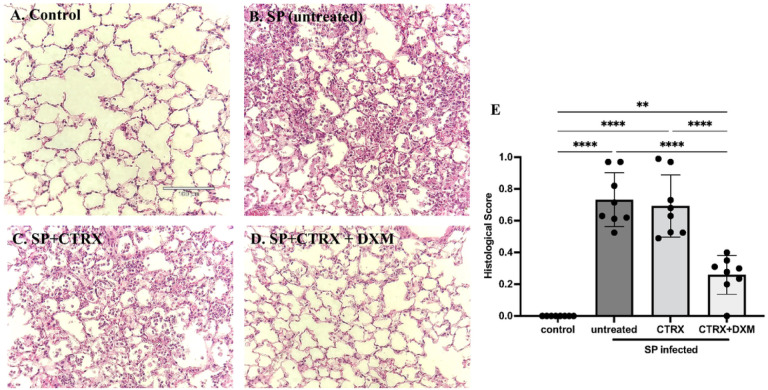
Dexamethasone improves histopathology in mice with pneumococcal pneumonia. (*A-D*) Representative images of lung histology; (*E*) Histological scores for each group. Compared with normal healthy control (*A*), the lungs of mice inoculated with *Streptococcus pneumoniae* (SP) had severe inflammation including the infiltration of neutrophils into the airspace, alveolar septal thickening, and alveolar edema fluid at 36 hours after infection (*B*). Treatment with ceftriaxone had little effect on the inflammation despite of its capacity to eliminate the bacteria (*C*). The combination of dexamethasone and ceftriaxone further reduced the inflammation (*D*) and significantly improved the histopathological score comparable to healthy control (*E*). n = 4/group. Data are shown as means ± SD. Statistical differences between groups were calculated with with one-way ANOVA followed by Tukey’s multiple comparison test **P < 0.01, ****P < 0.0001. ***Abbreviations*:** SP *streptococcus pneumoniae*; CTRX ceftriaxone; DXM dexamethasone.

As quantified by an investigator blinded to the experimental condition, when compared to healthy mice (Fig. 4A), the lungs of mice inoculated with *S. pneumoniae* had severe inflammation including the infiltration of neutrophils into the airspaces and alveolar septal thickening and alveolar edema fluid at 36 hours after infection (Fig. 4B). Treatment with ceftriaxone alone had no effect on histological markers of lung injury despite its capacity to eliminate bacteria (Fig. 4C). In contrast, the combination of ceftriaxone and dexamethasone significantly and markedly reduced histological markers of lung injury and inflammation and significantly improved the histopathological score compared to untreated controls and to ceftriaxone alone (Fig. 4D, E), findings that are consistent with the results of pulmonary edema, total protein concentration and white blood cells in the BAL.

#### Alveolar Epithelium – Functional and Biochemical Studies.

To test the effect of dexamethasone on the vectorial fluid clearance capacity of alveolar epithelium [28, 29], we measured alveolar fluid clearance 36 hours after infection. Alveolar fluid clearance was substantially impaired with pneumococcal pneumonia, but there was no significant difference between the untreated and other treatment groups (**Figure S5A**), indicating that the favorable effects of dexamethasone combined with antibiotics on pulmonary edema, hypoxemia and histology were explained by a reduction in the formation of pulmonary edema not by an increase in the rate of alveolar fluid clearance.

In addition, we measured BAL levels of RAGE, a type I epithelial cell injury marker [30, 31]. There was a significant increase in RAGE in BAL of pneumococcal infected mice (**Figure S5B**), indicating significant alveolar epithelial cell injury. However, the level of RAGE in BAL was not reduced by ceftriaxone or dexamethasone, a result that supports no significant effect of either treatment on the vectorial fluid transport capacity of the alveolar epithelium.

### Dexamethasone attenuates the inflammatory responses in the air spaces of mice with pneumococcal pneumonia

**Figure 5:**
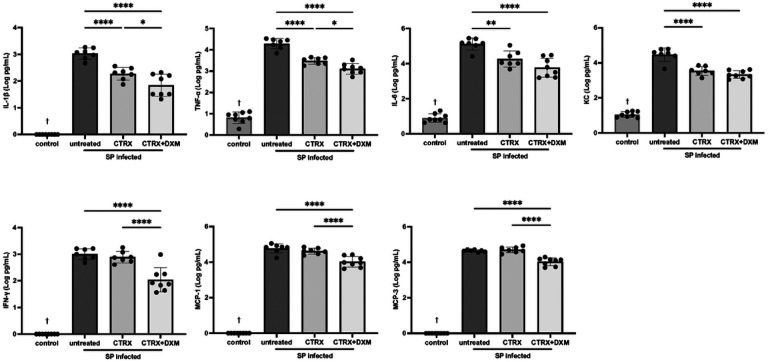
Dexamethasone attenuates the inflammatory response in mice with pneumococcal pneumonia. Inflammatory biomarkers including IL-1β, tumor necrosis factor-α (TNF-α), IL-6, keratinocyte chemoattractant (KC), interferon-γ (IFN-γ), monocyte chemoattractant protein-1 (MCP-1) and MCP-3 were significantly higher in infected mice compared with normal healthy control. Ceftriaxone reduced IL-1β, TNF-α, IL-6, and KC, but did not impact the levels of IFN-γ, MCP-1, and MCP-3. The combination of ceftriaxone and dexamethasone significantly attenuated all of these inflammatory cytokine and chemokine accumulation in the air spaces compared with untreated controls and significantly reduced IL-1β, TNF-α, IFN-γ, MCP-1, and MCP-3 as compared to ceftriaxone alone. n = 7–8/group. Data are shown as means ± SD. Statistical differences between groups were calculated with one-way ANOVA followed by Tukey’s multiple comparison test. *P < 0.05, **P < 0.01, ***P < 0.001, ****P < 0.0001. ^†^P < 0.05 compared with infected groups. ***Abbreviations*:** SP *streptococcus pneumoniae*; CTRX ceftriaxone; DXM dexamethasone; TNF-α tumor necrosis factor-α; KC keratinocyte chemoattractant; IFN-γ interferon-γ; MCP monocyte chemoattractant protein.

We measured cytokines and chemokines in BAL fluid using a multiplex assay to quantify the inflammatory responses with dexamethasone treatment for pneumococcal pneumonia. Selected cytokines and chemokines with the high relevance to lung injury are displayed in Fig. 5. IL-1β, TNF-α, IL-6, and IFN-γ and chemokines including KC, MCP-1, and MCP-3 were significantly higher in infected mice compared with normal healthy control mice (Fig. 5). Ceftriaxone reduced IL-1β, TNF-α, IL-6, and KC, but did not impact the levels of IFN-γ, MCP-1, and MCP-3. Importantly, the combination of ceftriaxone and dexamethasone significantly attenuated all of these inflammatory cytokine and chemokine accumulation in the air spaces compared with untreated controls and significantly reduced IL-1β, TNF-α, IFN-γ, MCP-1, and MCP-3 as compared to ceftriaxone alone. Additional biomarkers were measured, several of which showed a decrease in BAL concentration with dexamethasone and antibiotic treatment (**Figure S6**). These results indicate that dexamethasone in combination with appropriate antibiotics reduces the release of pro-inflammatory cytokines and chemokines in pneumococcal pneumonia.

## DISCUSSION

This study addresses several important clinically relevant gaps in our knowledge regarding bacterial pneumonia and the effects of corticosteroid treatment. First, prior to this work, limited data were available on the biological overlap of experimental pneumonia in mice compared to patients with pneumococcal pneumonia. Second, few studies have directly evaluated the mechanistic effects of corticosteroids in an experimental model of pneumococcal pneumonia, a particularly important issue in view of the recent clinical trials reporting improved outcomes in patients with severe community acquired pneumonia when treated with corticosteroids [11]. Finally, although patients recognized to have bacterial pneumonia are uniformly treated with antibiotics, animal models using bacterial pathogens do not routinely include the use of antibiotics. This study identifies several mechanisms by which dexamethasone decreases lung injury in a *S. pneumoniae* model and supports the biological relevance of this model to humans with community acquired pneumococcal pneumonia.

RNA sequencing identified several pathways that were dysregulated by *S. pneumoniae* infection in both humans and mice. We identified signatures of inflammasome activation, including IL1 signaling and NF-kB activation, and neutrophil degranulation in infected mice and humans. Transcriptomic analysis also identified several pathways modified by the addition of dexamethasone to ceftriaxone. While steroids decreased the expression of some pathways upregulated in *S. pneumoniae*-infected mice compared to controls, they also increased the expression of other pathways, suggesting the effects of steroids are more complex than simply reversing expression of dysregulated pathways. Notably, tracheal aspirate RNA sequencing from mechanically ventilated patients with *S. pneumoniae* infection identified similar gene expression changes to those observed in the mouse model, suggesting this experimental system replicates several biological features of clinical disease. In addition, some pathways that were modified by dexamethasone treatment in mice also were also modified in humans treated with steroids compared to those who were not.

The mouse studies of pneumococcal pneumonia demonstrated therapeutic benefits of dexamethasone on oxygenation, pulmonary edema, and lung histology when added to antibiotics. These results are consistent with recent clinical studies, in which early administration of dexamethasone reduced the duration of mechanical ventilation in ARDS patients and reduced the progression to mechanical ventilation in non-intubated patients with severe pneumonia [11, 32, 33]. Assessment of BAL fluid identified that the combination of antibiotics and dexamethasone reduced protein and leukocyte permeability into the distal air spaces. In contrast to the favorable effect on protein permeability, dexamethasone did not enhance alveolar fluid clearance, suggesting that the favorable effects of dexamethasone combined with antibiotics were explained by a reduction in the formation of pulmonary edema, not by an increase in the rate of alveolar fluid clearance.

Because of their immunosuppressive effects, steroids can impair host bacterial clearance [34]. In one mouse study using a very early administration of glucocorticoids (one hour after infection) [14], high dose dexamethasone increased bacterial burden in the lung. In contrast, dexamethasone did not impair host bacterial clearance in the current study, possibly because dexamethasone was given 20 hours after infection and at the same time antibiotic therapy was initiated. We selected this schedule in part to enhance the clinical relevance of the model, as patients with pneumonia usually come to medical attention when their infection has progressed enough to cause symptoms. These findings are consistent with the results of some recent clinical trials of steroids in COVID-19 ARDS [35, 36], non-COVID ARDS [33], and meta-analyses of steroid use in community-acquired pneumonia [37], in which steroid administration provided clinical benefit in patients with pneumonia of differing etiologies but was not associated with significantly higher rates of secondary infection.

Since dexamethasone has pleiotropic effects on the immune system [34], we investigated the impact of dexamethasone on the release of inflammatory cytokines and chemokines. The combination of dexamethasone and ceftriaxone attenuated the release of inflammatory cytokines in BAL fluid, including IL-1β, IL-6, TNF-α, and IFN-γ, and chemokines including KC, MCP-1, and MCP-3. These results are consistent with a prior study of corticosteroid treatment in ARDS patients [38]. Also, the combination of dexamethasone and ceftriaxone significantly reduced the release of IL-1β, TNF-α, IFN-γ, MCP-1, and MCP-3 compared with ceftriaxone alone. An experimental study using a mouse model of pneumococcal pneumonia reported that neutralization of IFN-γ accelerates recovery from lung injury [39]. Growing evidence suggest that MCP-1 is involved in lung inflammatory disorders [40]. The beneficial effect of dexamethasone on lung injury might be attributed in part through suppressing the release of these proinflammatory cytokines and chemokines. Transcriptomic analyses also identified additional pathways, including increased non-canonical NF-kB signaling and shifts in metabolic pathways, that may be important targets for future research.

This study has some limitations. First, the sample size from our clinical cohort is modest. Steroid treatment was non-randomly assigned and likely more commonly administered in sicker patients. In addition, our clinical respiratory tract samples were from tracheal aspirates, while our mouse experimental samples were from whole lung homogenate, which likely decreased our ability to detect differentially expressed genes overlapping between humans and mice. We were unable to identify individual genes that were significantly differentially expressed with steroids in humans. Despite this, we were able to detect changes at the pathway level that were shared between our experimental mouse model and the clinical samples. Second, our experimental studies in mice focused on the early phase of lung injury from pneumococcal pneumonia but did not address survival. This design made it possible to understand the earliest effects of steroid therapy on critical physiologic end points (oxygenation, lung fluid and protein balance), inflammatory responses, lung pathology, and bacterial burden. A recent clinical trial [11] reported a mortality benefit for corticosteroids in severe CAP, so we did not think that survival studies were as critical compared to in depth mechanistic analyses. Future studies will need to test the effects of steroids in other bacterial and viral pathogens in addition to the results from this study with *S. pneumoniae*.

## CONCLUSIONS

In summary, adjunctive therapy with both antibiotics and dexamethasone reduces the severity of hypoxemia, pulmonary edema, and histologic evidence of lung injury in experimental pneumococcal pneumonia compared to antibiotics alone, in part through attenuating the release of inflammatory cytokines and chemokines. Importantly, steroid therapy in combination with antibiotics does not increase bacterial burden in the early phase of pneumococcal pneumonia in mice. Transcriptomic profiling supports the clinical relevance of this mouse model, which replicates several features of pneumococcal pneumonia and steroid therapy in humans. In conclusion, steroid therapy has a sound mechanistic basis as an adjunctive treatment for severe community acquired bacterial pneumonia from *S. Pneumoniae* with the use of appropriate antibiotics.

## Data Availability

Sequencing data for murine experiments is available at GSE246398. Because some tracheal aspirates samples were collected under a waiver of informed consent, the UCSF IRB prohibits the public sharing of genomic data from the human subjects included in this study. Processed gene count data for humans and mice are available in Supplementary Data 1. Researchers interested in studying the genomic data from human subjects should contact the corresponding author (Hiroki.Taenaka@ucsf.edu).

## Abbreviations

ARDS: Acute respiratory distress syndrome
CAP: Community acquired pneumonia
ELW: Excess extravascular lung water
BAL: Bronchoalveolar lavage
RAGE: Receptor for advanced glycation
GSEA: Gene set enrichment analysis
WGCNA: Weighted gene co-expression network analysis
